# Female Mice are Protected against High-Fat Diet Induced Metabolic Syndrome and Increase the Regulatory T Cell Population in Adipose Tissue

**DOI:** 10.1371/journal.pone.0046057

**Published:** 2012-09-25

**Authors:** Ulrika S. Pettersson, Tomas B. Waldén, Per-Ola Carlsson, Leif Jansson, Mia Phillipson

**Affiliations:** 1 Department of Medical Cell Biology, Uppsala University, Uppsala, Sweden; 2 Department of Medical Sciences, Uppsala University, Uppsala, Sweden; University of Bremen, Germany

## Abstract

Sex differences in obesity-induced complications such as type 2 diabetes have been reported. The aim of the study was to pinpoint the mechanisms resulting in different outcome of female and male mice on a high-fat diet (HFD). Mice fed control or HFD were monitored for weight, blood glucose, and insulin for 14 weeks. Circulating chemokines, islet endocrine function and blood flow, as well as adipose tissue populations of macrophages and regulatory T-lymphocytes (T_reg_) were thereafter assessed. Despite similar weight (43.8±1.0 and 40.2±1.5 g, respectively), male but not female mice developed hyperinsulinemia on HFD as previously described (2.5±0.7 and 0.5±0.1 pmol/l, respectively) consistent with glucose intolerance. Male mice also exhibited hypertrophic islets with intact function in terms of insulin release and blood perfusion. Low-grade, systemic inflammation was absent in obese female but present in obese male mice (IL-6 and mKC, males: 77.4±17 and 1795±563; females: 14.6±4.9 and 240±22 pg/ml), and the population of inflammatory macrophages was increased in intra-abdominal adipose tissues of high-fat-fed male but not female mice. In contrast, the anti-inflammatory T_reg_ cell population increased in the adipose tissue of female mice in response to weight gain, while the number decreased in high-fat-fed male mice. In conclusion, female mice are protected against HFD-induced metabolic changes while maintaining an anti-inflammatory environment in the intra-abdominal adipose tissue with expanded T_reg_ cell population, whereas HFD-fed male mice develop adipose tissue inflammation, glucose intolerance, hyperinsulinemia, and islet hypertrophy.

## Introduction

Obesity is associated with a chronic and systemic low-grade inflammation believed to contribute to development of insulin resistance [1], which, together with β-cell defects are key signs of type 2 diabetes [2], [3]. The low-grade systemic inflammation observed during obesity is especially manifested in adipose tissue and associated with increased levels of inflammatory mediators such as tumor necrosis factor-α, interleukin-6 (IL-6) and murine keratinocyte-derived chemokine (mKC, the murine ortholog of human interleukin-8) in circulation as well as in intra-abdominal adipose depots [4], [5], [6]. The inflammatory markers originate from both adipocytes and inflammatory macrophages infiltrating the expanding adipose tissue [7], and induce pro-inflammatory transcription factors which interfere with the systemic actions of insulin, resulting in peripheral insulin resistance [8].

During development of type 2 diabetes, insulin-producing β-cells initially compensate for the insulin resistance by enhanced insulin secretion, resulting in high circulating insulin levels. Furthermore, islet blood flow has been shown to increase in rodent models early after onset of type 2 diabetes [9], [10]. Increased islet blood perfusion most likely results in amplified shear stress on intra-islet endothelium due to the concomitant capillary hypertension [11], which, if persisting for longer periods, is believed to contribute to impairment of islet function [12]. In fact, in studies of young ob/ob mice and GK rats, islet blood flow was increased early after diabetes onset, but reversed to hypoperfusion in elder animals [13], [14], demonstrating an association of islet blood flow alterations and deterioration of islet function in type 2 diabetes. To further compensate for the insulin resistance, human studies of the β-cell mass revealed that when exposed to increased metabolic demands, i.e. obesity and pregnancy, the β-cell mass increases [15], [16], [17]. Indeed, failure of this β-cell adaptation together with increased β-cell apoptosis has been suggested to be a contributing factor to the onset of type 2 diabetes [15], [18].

Obvious sex differences in the susceptibility to various diseases have been detected, even though the reasons for this are not completely clear. Estrogens, the major female sex hormone, are suggested to protect against development of the metabolic syndrome, and the prevalence of obesity, insulin resistance, and type 2 diabetes increases in post-menopausal women [19]. However, conflicting data on circulating levels of inflammatory markers in women and men during normal conditions and various diseases have been presented, and the concentrations of pro-inflammatory IL-6 have been shown to be higher in men than women, but also opposite data exist [6], [20], [21]. In addition, different studies indicate that estrogens may have pro- as well as anti-inflammatory effects [22]. C-reactive protein is elevated [23] or unaffected [24], [25] by estrogen stimuli, whereas monocyte chemoattractant protein-1 and soluble vascular cell adhesion molecule-1, two important regulators of the immune system, are decreased by estrogen treatment [25]. Sex differences on chemokine production, down-stream metabolic changes and effects on adipose tissue during obesity are not fully established.

In the current study, sex differences during onset of metabolic syndrome were investigated in female and male mice fed a high fat diet, with particular focus on adipose tissue inflammation and islet dysfunction.

## Materials and Methods

### Ethical Statement

All experiments followed the principles of laboratory animal care and were approved by the Swedish Laboratory Animal Committee, Uppsala (Permit Number C316/10).

### Animals

Female and male C57Bl/6 mice of 11–12 weeks of age (B&K Universal, Scanbur, Stockholm, Sweden), housed in the animal facility under standardized conditions had free access to water and pelleted control food (Type R36, Lantmännen, Kimstad, Sweden) or HFD (60% kcal from fat, D12492; Research diets, New Brunswick, NJ, USA).

### Plasma Glucose Concentrations and Tolerance Tests to Glucose and Insulin

Body weight and plasma glucose concentrations were followed during 14 weeks of diet intake. Intra-peritoneal glucose tolerance tests (GTT) and insulin tolerance tests (ITT) were performed on non-fasted animals after 14 weeks, two days prior experiments. Plasma glucose concentrations were measured in blood from the tail using test reagent strips (detection range 1.1–27.8 mmol/l; Freestyle, Abbott, Stockholm, Sweden) prior to, and 15, 30, 60, and 120 minutes after intraperitoneal injection of 2 g/kg body weight D-glucose (300 mg/ml; Fresenius Kabi, Uppsala, Sweden) for the GTT, and the same time points for the ITT, in addition to at 90 minutes after intraperitoneal injection of 2 U/kg body weight insulin (Novorapid, Novo Nordisk A/S, Copenhagen, Denmark).

### Serum Levels of Inflammation Markers, Insulin, and Lipids

Mouse Proinflammatory 7-Plex Assay Ultra-Sensitive Kit (Mesoscale, Gaithersburg, MD, USA) was used to detect serum levels of interleukin-6 (IL-6), IL-10, IL-1β, IL-12p70, IFN-γ TNF-α and mKC, but only reliable values of IL-6, IL-10 and mKC could be generated using mouse serum. Serum levels of insulin (Rat Insulin ELISA™; Mercodia, Uppsala, Sweden), as well as triglycerides and cholesterol concentrations in serum were measured (Architect c4000 Abbott Laboratories, Abbott Park, IL, USA) after the experiments.

### Blood Flow Measurements

Mice were anesthetized by intraperitoneal injections of avertin (2.5% [vol/vol] solution of 10 g 97% [vol/vol] 2,2,2-tribromoethanol [Sigma-Aldrich, St. Louis, MO, USA] in 10 ml 2-methyl-2-butanol [Kemila, Stockholm, Sweden]) and placed on a heating pad to maintain body temperature at ∼38°C. Animals were tracheostomized to facilitate respiration. Polyethylene catheters were inserted into right carotid artery for blood pressure measurements (PDCR 75/1; Druck; Groby, UK) and in the left femoral artery for blood sampling.

After a 15-minute resting period, blood flow measurements were performed using a microsphere technique as previously described [26]. Briefly, ∼1.5×10^5^ black non-radioactive microspheres (diameter 10 µm, EZ-Trac™; Triton microspheres, San Diego, CA, USA) were injected through the carotid catheter for 5 seconds. For a total of 60 seconds, arterial blood was collected by free flow from the catheter in the femoral artery. The exact withdrawal rate was confirmed by weighing the sample. Pancreas, both adrenal glands and samples (∼40 mg) from the colon, duodenum, and kidney were removed and weighed. The number of microspheres in the samples, including pancreatic islets was counted as previously described after visualization of islet microspheres by a freeze-thawing technique [27]. The total number of islets and the total number of perfused islets were counted. The fractional islet mass was estimated using a grid and islet diameter was measured using ImageJ (rsbweb.nih.gov/ij/). Organ blood flow (ml×min^−1^×g^−1^ tissue) values were calculated using the formula Q_org_ = Q_ref_×N_org/_N_ref_ were Q_org_ is the organ blood flow, Q_ref_ is withdrawal rate of the reference sample, N_org_ the number of microspheres in the organ and N_ref_ the number of microspheres in the reference sample. Islet perfusion was expressed as µl×min^−1^×g^−1^ pancreas or as µl×min^−1^×mg^−1^ islet weight. Adequate mixing of microspheres in the circulation was confirmed by comparing the blood flow values of the adrenal glands, which had to be <10% within each animal.

### Islet Isolation, Insulin Release, and Insulin Content

Islets were isolated by collagenase digestion and cultured for two days as previous described [28]. Triplicate groups of ten islets were placed in glass vials containing 0.25 ml of Krebs-Ringer bicarbonate buffer supplemented with 2 mg/ml bovine serum albumin (fraction V; MP Biomedicals, Aurora, OH, USA). During the first hour islets were incubated in 1.7 mmol/l glucose medium in 95% air/5% CO_2_ at 37°C. One hour later, medium was removed and replaced with media containing 16.7 mmol/l glucose followed by a second 1-hour-incubation. After incubations, triplicates were pooled and homogenized. A fraction of the homogenate was mixed with acid-ethanol (0.18 M HCl in 95% (vol/vol) ethanol) from which insulin was extracted overnight at 4°C. Insulin contents in the incubation medium and homogenates were determined using Mesoscale insulin assay kit (Mesoscale Discovery) and DNA content was determined using a Nanodrop™ 2000c spectrophotometer (Thermo-Scientific, Wilmington, DE).

### Macrophage Content in Visceral Adipose Tissues

Twelve µm thick cryosections of gonadal and mesenteric adipose tissue were stained for macrophage content using primary antibodies: rat anti-mouse F4/80 (1∶200, eBioscience, San Diego, CA, USA), Alexa Fluor488-conjugated MGL-1 (macrophage galactose-type C-type lectin 1/CD301, 1∶200, AbD Serotec, Düsseldorf, Germany). Slides were washed before incubation with secondary antibody NL577 goat anti-rat IgG (1∶500, R&D Systems, Minneapolis, MN, USA). All slides were co-stained with Hoechst33342 (Invitrogen, Stockholm, Sweden). Images were captured using a Nikon confocal microscope (Nikon C-1 with Plan Apo VC 20x/0.75 objective) and analyzed using ImageJ. The mean value of three images per adipose tissue depot was used in one observation.

### Regulatory T-cell Content in Visceral Adipose Tissues

The stromal vascular fraction of gonadal and mesenteric adipose tissue was isolated from female and male mice fed a control or HFD. Digestion of adipose tissue was performed for 45 minutes in 37°C using a collagenase digestion buffer with continuous mixing. The suspension was filtered through a 250 µm nylon filter and put on ice for 30 minutes. The bottom layer was filtered through a 40 µm filter before incubation with erythrocyte lysis buffer for 10 minutes. The fraction was stained using FITC-conjugated CD4 antibody and APC-conjugated Foxp3-antibody (eBioscience) before FACS analysis.

### RNA Isolation from Adipose Tissue and Real-Time qPCR

Total RNA was extracted from frozen gonadal adipose tissue with Trizol (Invitrogen, Carlsbad, CA, USA) according to the manufacturer’s protocol, and RNA concentrations were measured on a Nanodrop™ 2000c spectrophotometer (Thermo-Scientific). To synthesize cDNA, 2 µg RNA from each sample was reverse-transcribed with a High Capacity cDNA kit (Applied Biosystems, Foster City, CA, USA). To measure Foxp3 and 18S mRNA, 2 µl of each cDNA sample were loaded in duplicate in TaqMan Gene Expression mastermix (Applied Biosystems) together with a MGB probe targeting Foxp3 (Mm00475162_m1) or 18S (Hs99999901_s1) (Applied Biosystems) on a MyIQ2 Real Time PCR System (Bio-Rad Laboratories, Sundbyberg, Sweden). According to the comparative threshold method (Ct-method), Foxp3 mRNA levels were normalized to 18S mRNA levels (ΔCt). The average ΔCt value from female mice on control diet was used as the 100% reference (ΔΔCt) for Foxp3 mRNA expression. The formula 2^−ΔΔCt^ was applied to convert the logarithmic ΔΔCt values to linear values.

### Statistical Analysis

All values are expressed as mean ± SEM. One way ANOVA was used for the statistical analysis, and Fischer’s LSD as a post hoc test using SigmaStat 3.5 (Systat Software, Richmond, VA, USA). Statistical significance was set to P<0.05.

## Results

### Sex Differences in Development of Hyperinsulinemia and Insulin Resistance Despite Similar Weight Gain after High-fat Diet

**Figure 1:**
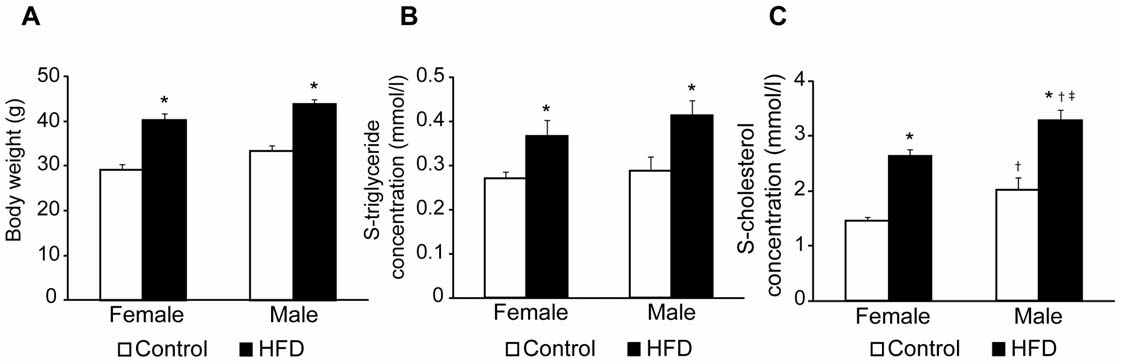
Body weight and adiposity in female and male mice on control or high-fat diet (HFD). **A**, Body weight in control female (n = 9), HFD female (n = 8), control male (n = 7), and HFD male (n = 7) mice. Serum concentrations of triglycerides (**B**) and cholesterol (**C**) in female control (n = 7), female HFD (n = 8), male control (n = 6), and male HFD (n = 7) mice. Data are means ± SEM. *P*<0.05 compared with control (*), compared with female control (†), and compared with female on HFD (‡).

**Figure 2:**
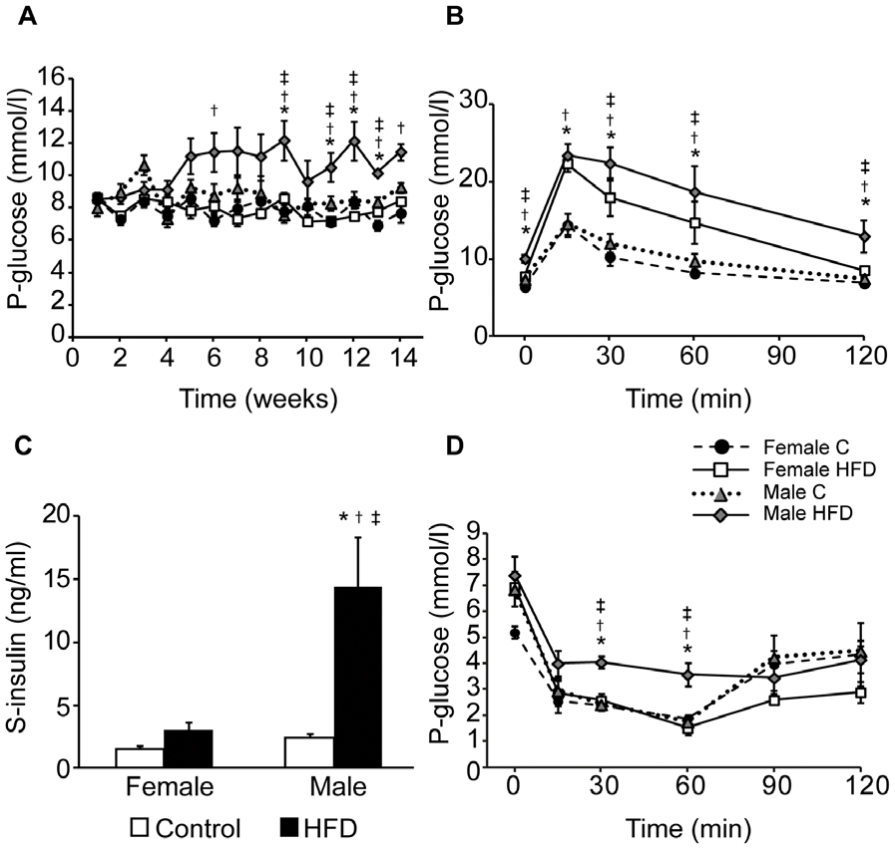
Effects of a high-fat diet (HFD) on circulating glucose and insulin metabolism. **A**, Plasma glucose concentrations during 14 weeks of control or HFD in female (n = 7 and 4) and male (n = 4 and 4) mice. **B**, Glucose tolerance test in non-fasted control female (n = 12), HFD female (n = 10), control male (n = 11), and HFD male (n = 10) mice after 14 weeks of control or HFD. **C**, Serum insulin concentrations in control female (n = 9), HFD female (n = 8), control male (n = 7), and HFD male (n = 7) mice after 14 weeks on control or HFD. **D**, Insulin tolerance test in non-fasted control female (n = 4), HFD female (n = 4), control male (n = 4), and HFD male (n = 4) mice after 14 weeks on control or HFD. Data are means ± SEM. *P*<0.05 compared with control (*), compared with female control (†), and compared with female on HFD (‡).

High-fat diet was used to induce metabolic syndrome, and firstly the stage of the disorder had to be defined. Female and male mice on HFD increased similarly in weight during the studied period of 14 weeks, which was significantly more than mice on control diet (Figure 1A). Circulating levels of serum triglycerides and cholesterol concentrations were increased in high-fat-fed female and male mice compared to control mice (Figure 1B–C). However, male mice had higher serum cholesterol concentrations compared to female mice, which increased further by high-fat-feeding (Figure 1C). Plasma glucose concentrations increased in high-fat-fed male mice over time compared to mice on control diet, whereas plasma glucose levels of high-fat-fed female mice remained constant (Figure 2A). After 14 weeks of HFD, the glucose tolerance was impaired compared to age-matched control mice. This impairment was more pronounced in HFD-fed male mice (Figure 2B). In addition, hyperinsulinemia consistent with insulin resistance was detected in high-fat-fed male mice, whereas female mice on HFD had normal serum insulin concentrations despite a similar increase in body weight as the male mice on HFD (Figure 2C). Further, only male mice on HFD showed impaired insulin tolerance 30 and 60 minutes after an ITT compared to all other groups (Figure 2D), consistent with established insulin resistance in male but not female mice.

### High-fat Diet Increases Islet Size in Male but not Female Mice

**Figure 3:**
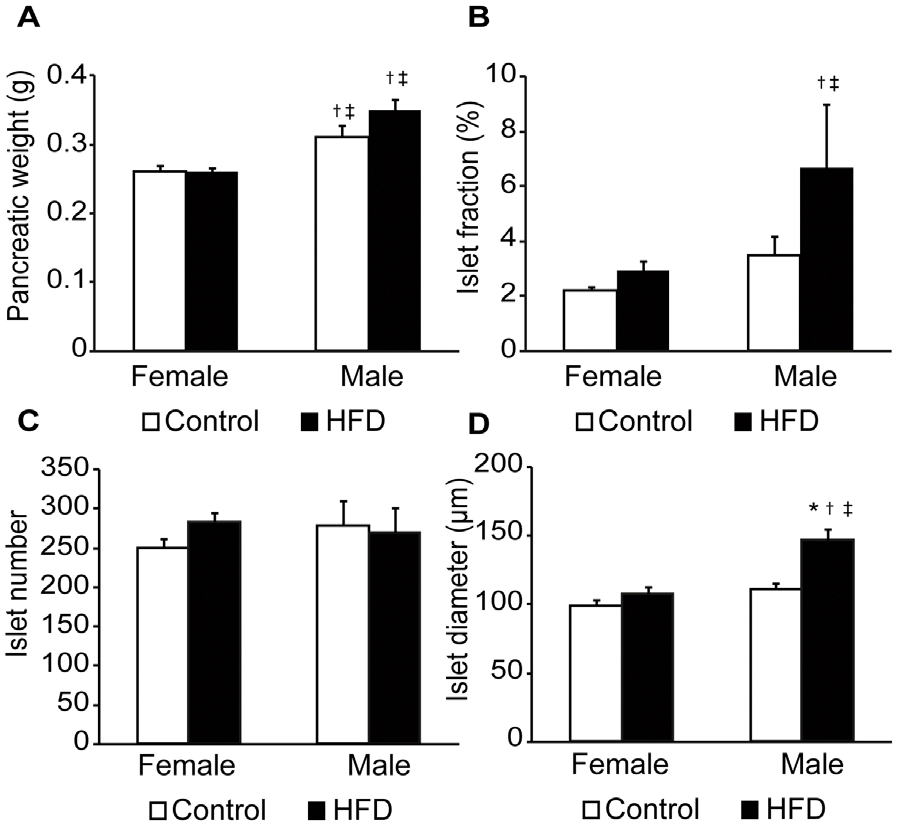
Effects on pancreas and islets of a high-fat diet (HFD) in female and male mice. Pancreatic weight **(A**), islet fraction (**B**), total number of islets (**C**), and islet diameter (**D**) in control female (n = 9), high-fat diet (HFD) female (n = 8), control male (n = 7), and HFD male (n = 7) mice after 14 weeks of diet. Data are means ± SEM. *P*<0.05 compared with control (*), compared with female control (†), and compared with female on HFD (‡).

We then wanted to investigate if the established hyperinsulinemia had resulted in compensations at the level of islets of Langerhans. The pancreatic weight was found to be higher in male mice than in female mice irrespective of diet (Figure 3A). The fraction of islets was increased in male mice after HFD when compared to high-fat-fed female mice, whereas no differences were seen between the control-fed animals (Figure 3B). This observation was not due to increased islet number, as the total number of islets in the groups did not differ between sexes or diets (Figure 3C). Instead, the mean islet diameter in the sections was increased in the high-fat-fed male mice compared to all other groups, which suggest that these islets become hypertrophic in response to HFD (Figure 3D).

### Blood Flow Differences in Female and Male Mice on High-fat Diet

**Figure 4:**
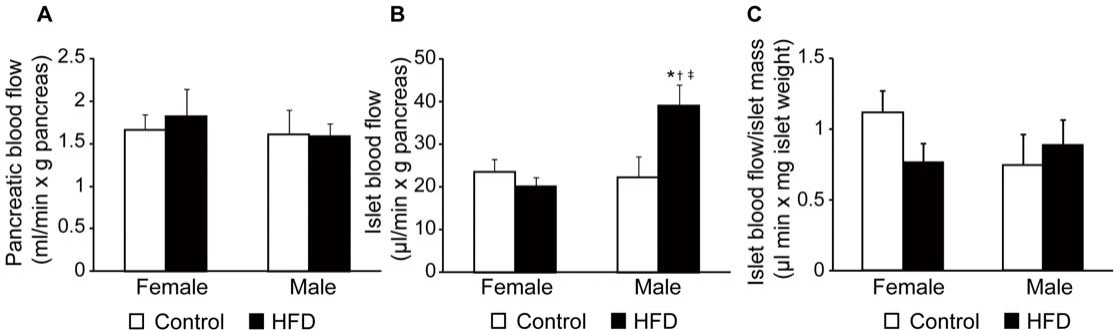
Effects of a high-fat diet (HFD) on pancreas blood perfusion. Pancreatic blood flow (**A**), islet blood flow per g pancreas (**B**), and islet blood flow per islet mass (**C**) in control female (n = 9), HFD female (n = 8), control male (n = 7), and HFD male (n = 7) mice after 14 weeks on diet. Data are means ± SEM. *P*<0.05 compared with control (*), compared with female control (†) and compared with female on HFD (‡).

To investigate if 14 weeks of HFD affect the blood flow in the pancreas and its islets, microsphere injections were performed. No differences in blood pressure could be detected between anesthetized control female, HFD female, control male, and HFD male mice (71±3, 72±2, 78±4, and 83±12 mmHg, respectively). While blood flow was in most cases higher in reference organs of control and high-fat-fed male compared to control female mice (Table 1), no sex difference or impact of HFD was detected for the total pancreatic blood flow (Figure 4A). However, blood flow to the islet organ (islet blood flow per g pancreas) was significantly increased in the high-fat-fed male mice, whereas all other groups showed similar islet blood flow levels (Figure 4B). When islet blood flow was expressed as blood flow per islet mass to compensate for the increased islet mass in high-fat-fed male mice (Figure 3B), no differences were seen between the groups (Figure 4C).

### Normal Islet Function in Mice Fed a High-fat Diet

**Figure 5:**
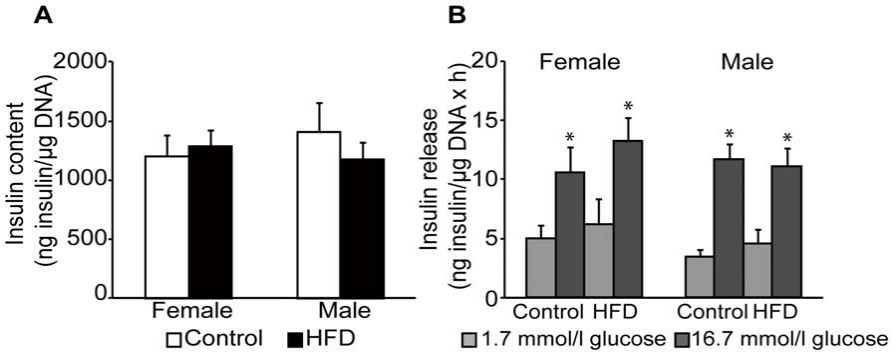
Effects on islet functionality by a high-fat diet (HFD) for 14 weeks. Insulin content (**A**) and insulin release in 1.7 or 16.7 mM glucose (**B**) in control female (n = 5), HFD female (n = 6), control male (n = 5), and HFD male (n = 6) mice of isolated pancreatic islets after 48h-culture expressed as ng/µg DNA. Data are means ± SEM. *P<0.05 compared with control.

To investigate if the high-fat-fed mice had developed impaired islet function, total insulin content as well as glucose-stimulated insulin release was studied *in vitro* from isolated islets. Islet insulin contents were similar in all groups (Figure 5A), and no differences in insulin release during low (1.7 mmol/l) and high (16.7 mmol/l) glucose incubation were detected in either control or high-fat-fed mice (Figure 5B). Thus, 14 weeks of HFD resulted in an early stage of metabolic syndrome in male mice demonstrated by hyperinsulinemia and islet hypertrophy but still intact islet function in respect to insulin content and insulin secretion, while female mice were apart from the increased body weight still largely unaffected.

**Table 1:** Organ blood flow.

Organ blood flow						
	Female			Male		
	Control (n = 9)		HFD (n = 8)	Control (n = 7)		HFD (n = 7)
Colonic blood flow (ml×min^−1^×g^−1^)	1.6±0.4		3.1±0.7	3.4±0.7		2.1±0.5
Duodenal blood flow (ml×min^−1^×g^−1^)	3.7±0.5		4.8±0.8	7.9±0.6*^†^		7.1±1.2*
Renal blood flow (ml×min^−1^×g^−1^)	4.4±1.0		5.2±0.9	8.8±0.9*		6.2±1.2
Adrenal blood flow (ml×min^−1^×g^−1^)	17.3±2.4		19.0±4.0	36.8±6.2*		39.8±12.1*^†^

Values are means ± SEM. P<0.05 compared with female control (*), compared with female HFD (†).

### Inflammatory Sex Differences in High-fat-fed Mice

**Figure 6:**
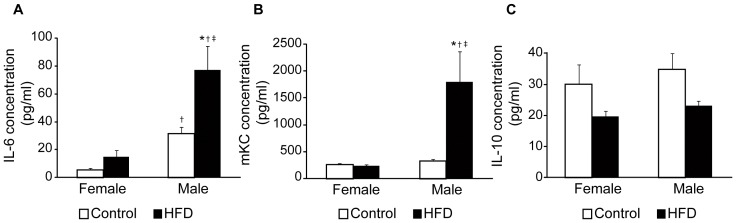
Serum concentrations of inflammatory markers after 14 weeks of control or high-fat diet (HFD). A. Serum IL-6 concentrations in control female (n = 4), HFD female (n = 5), control male (n = 6), and HFD male (n = 6) mice. **B**. Serum mKC concentrations in control female (n = 6), HFD female (n = 7), control male (n = 7), and HFD male (n = 7) mice. **C**. Serum concentrations of anti-inflammatory IL-10 in control female (n = 8), HFD female (n = 8), control male (n = 8), and HFD male (n = 7) mice. Data are means ± SEM. *P*<0.05 compared with control (*), compared with female control (†) and compared with female on HFD (‡).

Insulin resistance is associated with low-grade systemic inflammation, and circulating serum inflammatory markers were therefore measured. Increased levels of the chemokines IL-6 and mKC were detected in high-fat-fed male mice compared to all other groups (Figure 6A–B). There was also a tendency towards decreased levels of the anti-inflammatory chemokine IL-10 in male mice fed a HFD (Figure 6C, males: *P* = 0.063, females: *P* = 0.135).

### High-fat-fed Male but not Female Mice Show Increased Number of F4/80+MGL-1- Macrophages in Visceral Adipose Tissue

**Figure 7:**
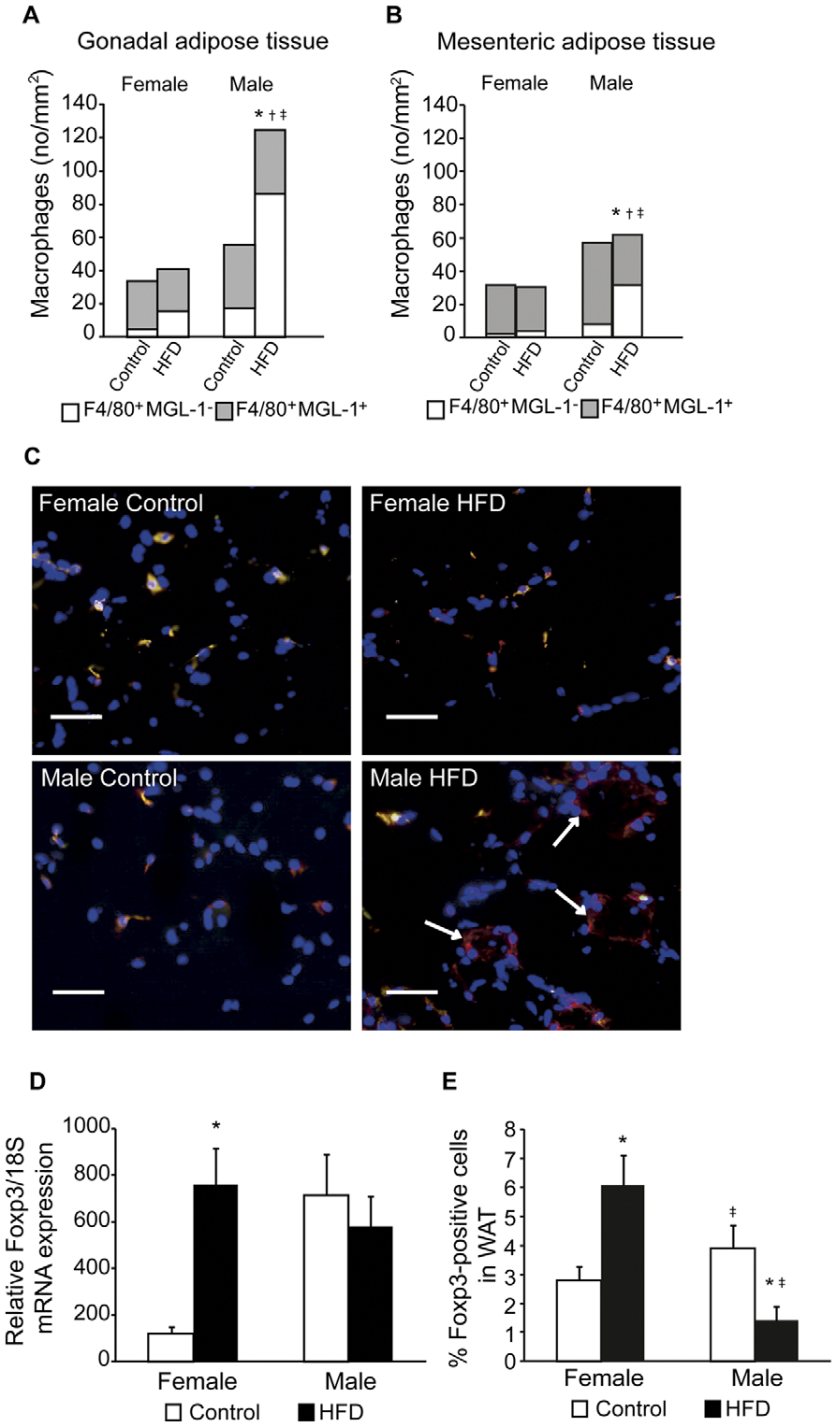
The effect of a high-fat diet (HFD) on the leukocyte populations visceral adipose tissues. Number of leukocyte subtypes per mm^2^ of gonadal visceral adipose tissue (**A**) and mesenteric adipose tissue (**B**) in control female (n = 4 and 6, respectively), HFD female (n = 7 and 6), control male (n = 5 and 6) and HFD male (n = 8 and 6) mice. F4/80^+^MGL-1^+^ are resident macrophages (M2-like) and F4/80+MGL-1- are inflammatory macrophages (M1-like). *P*<0.05 for the F4/80^+^MGL-1^−^ macrophages compared with control (*), compared with female control (†) and compared with female on HFD (‡). **C**. Representative images of stained macrophages in gonadal adipose tissue of control female, HFD female, control male, and HFD male mice. M2-like macrophages are double positive for MGL-1 (Alexa Fluor488, green) and F4/80 (NL557, red), while M1-like macrophages are only positive for F4/80 (red). Nuclei are stained blue (Hoechst33342). Scale bar equals 50 µm and arrows mark crown-like structures. **D**. Foxp3 mRNA levels in gonadal adipose tissue (n = 6 in all groups). Foxp3 Ct-values were normalized to 18S and the mean of the linearized expression values from female mice on control diet were set as 100%. **E**. Percentage of Foxp3-positive cells in gonadal and mesenteric adipose tissue in control female (n = 10), HFD female (n = 10), control male (n = 12), and HFD male (n = 13) mice after 14 weeks on HFD. All data are means ± SEM. *P*<0.05 compared with control (*), compared with female control (†) and compared with female on HFD (‡).

Since inflammatory chemokines in serum are believed to originate to a substantial degree from visceral adipose tissues [29], [30], the macrophage subtypes in different visceral adipose depots were investigated using immunohistochemistry. F4/80 was used as a general macrophage marker and MGL-1 as a marker for resident macrophages of the non-inflammatory M2-like subtype, as previously described by others [8]. F4/80^+^, MGL-1^−^ macrophages were considered as classical, inflammatory M1-like macrophages. The numbers of double positive F4/80^+^MGL-1^+^ (referred to as M2-like) macrophages were similar in all groups in the gonadal and mesenteric adipose tissue (Figure 7A and 7B). In contrast, the numbers of M1-like F4/80^+^MGL-1^−^ macrophages was significantly increased in the male mice fed a HFD in both gonadal and mesenteric adipose tissues (Figure 7A–C). These M1-like macrophages were detected to form crown-like structures surrounding adipocytes in male mice on HFD (Figure 7C), as previously described [31].

### Female Mice on High-fat Diet Increase the Expand Population of Foxp3-positive Regulatory T-lymphocytes

The population of anti-inflammatory regulatory T-lymphocytes (T_reg_) was recently reported to decrease in the visceral adipose tissues of obese mice and men [32], but eventual sex differences have not been studied. To investigate if HFD differently impact the adipose T_reg_ population in male and female mice, mRNA levels of the T_reg_ specific transcription factor Foxp3 as well as the fraction of Foxp3^+^ cells were measured in gonadal and mesenteric adipose tissue. Interestingly, Foxp3 mRNA increased 6.6-fold in female mice on HFD (Figure 7D). However, no up-regulation in response to HFD could be detected in male mice since Foxp3 mRNA levels were similar irrespective of diet. To investigate if the mRNA observations correspond to amount of Foxp3^+^ T_reg_ cells, single cell suspensions of gonadal and mesenteric adipose tissues were analyzed by cell sorting. Indeed, while T_reg_ numbers were decreased in male mice on HFD (Figure 7E), female mice on HFD increased the population of T_reg_ in the adipose tissue.

## Discussion

In the current study, obesity was induced in male and female mice by a high-fat diet. Despite similar weight gain, several sex differences were detected since male but not female mice developed visceral inflammation, glucose intolerance, insulin resistance, and hyperinsulinemia, as well as subsequent adaptations at the level of the insulin-producing islets. In complete contrast to observations made in male mice, the expanded visceral adipose tissue of female mice sustained a non-inflammatory nature with increased population of T_reg_, while withstanding recruitment of inflammatory macrophages.

Obesity, a result of positive energy balance, increases the risk for developing type 2 diabetes. In this study, obesity was induced by a diet resembling a presumed Western diet with high levels of fat and carbohydrates. Irrespective of sex, mice fed this HFD increased in body weight and circulating triglyceride concentrations compared to mice on control diet. In addition, plasma concentrations of glucose and cholesterol as well as serum insulin levels were increased in male mice on HFD, which, together with the observed impaired tolerance to glucose and insulin, indicate development of insulin resistance. The high-fat-fed female mice increased as much in weight as the male mice but did not develop any of the inflammatory parameters and did not differ from mice on control diet except for a slightly impaired response to glucose challenge.

Sex differences in the prevalence of metabolic diseases have previously been suggested in humans. Estrogens were demonstrated to be protective against the metabolic syndrome and type 2 diabetes in rodents [33], and the prevalence of metabolic diseases such as obesity, insulin resistance and type 2 diabetes dramatically increases post-menopause in women [19]. Treatment with 17β-estradiol or selective agonist binding estrogen receptor-α to glucose-intolerant and hyperinsulinemic female ob/ob mice [34] or ovariectomized mice on HFD [35] improved glucose tolerance and decreased insulin resistance [34], [35], but had no effect on insulin secretion [34]. These results indicate peripheral effects of estrogen rather than direct effects on islets. However, some animal studies suggest that estrogen directly improved β-cell function, through binding to its receptor in rat islets [36] and thereby stimulating insulin release [37]. In addition, controversies regarding the anti- or pro-inflammatory actions of estrogens have been reported in both animals and humans [22]. Circulating levels of IL-6 has been found to be higher in men than in women [20], but the opposite was reported for obese women [21] and for women after intake of a high-fat meal [24]. Moreover, serum levels of the human inflammation marker C-reactive protein were either increased [23] or unaffected [24], [25] by estrogens. Further, estrogens were shown to inhibit IL-6 production by liver Kupffer cells in mice [38]. During obesity, increased circulating levels of different cytokines have been reported [1], [4], [39]. For instance, IL-6 production by monocytes and macrophages in adipose tissue increased during obesity [40], and is believed to contribute to development of insulin resistance in both humans and animals [7], [41], [42], [43], [44], [45]. The mouse homologue of human IL-8, mKC, originates from the stromal vascular fraction of adipose tissue and is increased in obese humans as well as mouse models of obesity [5], [46]. In the current study, elevated serum levels of both IL-6 and mKC were detected in high-fat-fed male, but not female, mice despite similar weight gain. This indicates that under the current settings, female mice are either resistant to, or have a delayed development of low-grade systemic inflammation in response to HFD-induced obesity.

It is generally believed that the low-grade systemic inflammation associated with obesity and the metabolic syndrome in humans originates from the expanded depots of visceral adipose tissue. Indeed, in the present study, increased amount of pro-inflammatory macrophages (F4/80^+^MGL-1^−^) was detected in adipose tissue from high-fat-fed male, but not female mice. These macrophages formed crown-like structures surrounding dead adipocytes present in adipose tissue, structures that could not be detected in female mice on HFD. These structures have been shown to consist of fused macrophages and lipid droplets forming multinucleate giant cells commonly seen in inflammatory adipose tissue [31]. However, manifest inflammation is a consequence of pro-inflammatory signals dominating the anti-inflammatory signals. The recent identification of different leukocyte subtypes with anti-inflammatory functions has lead to increasing interest in their involvement to prevent or modulate inflammation [47]. Alternatively activated M2-like macrophages are important in maintaining adipose tissue functions [48], and the amount of cells remained unchanged by 14 weeks of HFD in the current study.

It was recently shown that anti-inflammatory Foxp3+ T lymphocytes, T_reg_, are involved in adipose tissue homeostasis and that these cells decrease in number when mice or humans became obese [32], [49], [50], [51] due to suppressed differentiation by the inflammatory milieu [32]. In agreement with these studies, decreased numbers of adipose tissue T_reg_ cells were detected in high-fat-fed male mice in the present study. In contrast, female mice increased the population of anti-inflammatory T_reg_ in the expanding adipose tissues in response to HFD, as demonstrated by levels of Foxp3 mRNA as well as cell sorting of single cell suspensions, which might account for the absence of low-grade inflammation in these mice. The ability to endogenously expand the T_reg_ population in enlarged adipose tissues is an attractive approach to counteract inflammation and development of metabolic syndrome and has to our knowledge not previously been described. Intriguingly, a recent study comparing T cell populations in the circulation of obese and lean persons reported increased CD4^+^ T cells that were skewed towards the T_reg_ phenotype in the former group [52]. However, this study excluded persons with overt type 2 diabetes and liver enzyme abnormalities, and all of the remaining obese persons were indeed females (lean: 9 females of 11), which agrees with our observation of increased T_reg_ populations in adipose tissue of obese female mice. In contrast, another study in obese, insulin resistant patients, demonstrated decreased gene expression of Helios, a natural T_reg_ marker, in the visceral adipose tissue compared to lean individuals, whereas the FOXP3 gene expression was similar. However, the sexes of these persons are not reported, which precludes further comparisons to other studies. In the same study, depletion of the T_reg_ population in *db/db* mice was demonstrated to impair insulin sensitivity, which could be improved by adoptive T_reg_ transfer [53]. Even though the mechanisms behind the observed gender differences are still unknown, involvement of estrogens seems plausible. Ultimately, this phenomenon is likely to explain the lack of signs of hyperinsulinemia and islet hypertrophy in female mice in the current study. The disability of male mice to expand the population of anti-inflammatory cells in response to obesity might result in increased susceptible to adipose tissue inflammation and concomitant glucose intolerance.

An increased islet blood flow has in rodents been detected early after onset of obesity and type 2 diabetes and is believed to contribute to β-cell dysfunction [10], [13], [14]. The current study demonstrates that obesity without concomitant hyperinsulinemia and low-grade systemic inflammation did not induce blood flow increase since islet blood flow in high-fat-fed female mice was similar to mice on control diet. In contrast, high-fat-fed male mice, which developed both hyperinsulinemia and low-grade inflammation, demonstrated increased blood flow to the whole islet organ without any effects on pancreatic blood flow. The increased islet blood flow was paralleled with and merely reflected an increased islet mass. Augmentation of islet mass in high-fat-fed male mice was solely due to islet hypertrophy and not islet neogenesis, since the number of islets did not change. In previous studies, young male mice fed a HFD for 8 weeks increased their β-cell mass in order to stay normoglycemic [54], which appeared to be caused by increased islet sized rather than by formation of new islets [55], [56]. When islet functionality was assessed, similar insulin content and glucose-stimulated insulin release was revealed for islets obtained from animals of the different groups after compensation for observed differences in islet size. These results demonstrate that male but not female mice receiving HFD for 14 weeks develop hyperinsulinemia, alterations of total islet blood flow and islet size without substantial changes in β-cell function.

In conclusion, several sex differences were detected in HFD-fed mice despite similar weight gain. Female mice did not develop low-grade systemic inflammation, most probably due to the ability to expand the population of anti-inflammatory T_reg_ in the visceral adipose tissues and thereby counteract recruitment of M1-like macrophages and concomitant release of pro-inflammatory agents. In contrast, male mice developed pronounced hyperinsulinemia consistent with insulin resistance, high levels of circulating inflammatory markers, increased M1-like population and as a possible consequence, hypertrophic islets.
